# Assessment of allergen cross-reactivity

**DOI:** 10.1186/1476-7961-5-2

**Published:** 2007-02-09

**Authors:** Rob C Aalberse

**Affiliations:** 1 Sanquin Research and Karl Landsteiner Laboratory, Academic Medical Center, University of Amsterdam, Amsterdam, The Netherlands; 2 Department of Immunopathology, Sanquin Research, Plesmanlaan 125, NL, 1066 CX Amsterdam, The Netherlands

## Abstract

The prediction of allergen cross-reactivity is currently largely based on linear sequence data, but will soon include 3D information on homology among surface exposed residues. To evaluate procedures for these predictions, we need ways to quantitatively assess actual cross-reactivity between two allergens. Three parameters are mentioned: 1) the fraction of the epitopes that is cross-reactive; 2) the fraction of IgE that is cross-reactive; 3) the relative affinity of the interaction between IgE and the two allergens. This editorial briefly compares direct binding protocols with the often more appropriate reciprocal inhibition protocols. The latter type of protocol provides information on symmetric versus asymmetric cross-reactivity, and thus on the distinction between complete (= sensitising) allergens versus incomplete, cross-reacting allergens. The need to define the affinity threshold of the assay and a caveat on the use of serum pools are also discussed.

## 

In a paper recently published in this Journal, the question was raised whether a fungus considered for biological pest control (Beauvaria bassiana) could elicit allergic reactions due to cross-reactive IgE antibodies induced by allergens from known allergenic fungi [1]. Based on homology in the amino acid sequence, four potentially cross-reactive proteins were cloned, expressed in E coli and tested for IgE (cross)reactivity using sera from patients with known fungal allergies. Support for (cross)reactivity was found for two of these four proteins. The two (cross)reactive proteins had the highest sequence homology to known allergens: the enolase was 85% sequence-identical to the Alternaria enolase known as Alt a 6 and the aldehyde dehydrogenase was 71% sequence-identical to the Alternaria dehydrogenase Alt a 10. The two proteins with no demonstrable (cross)reactivity had sequence identities of 51 and 60% to two Aspergillus fumigatus proteins. It is tempting to conclude that this result supports the notion that sequence identity is a useful predictor of cross-reactivity.

A few comments on the prediction on cross-reactivity as illustrated by this study. As the authors point out, the number of sera used to test for cross-reactivity was small (N = 20, tested as 10 pools of 2; a caveat on the use of serum pools will be discussed later). Moreover, the clinical history of the patients is not specified, which is particularly relevant in view of the quite distinct modes of allergen exposure in case of invasive aspergillosis as compared to airborne Alternaria. IgE reactivity was investigated by immunoblotting of crude E. coli extracts as the source of the recombinant proteins, which is useful but not ideal since it is known to be inefficient for some allergens. Lastly, as discussed in more detail below, cross-reactive potential, particularly in case of polyclonal antibodies, is more reliably assessed by inhibition tests than by direct binding tests.

## Cross-reactivity and allergenicity

For various reasons, often related to regulatory safety issues, a discussion is ongoing on prediction of allergenicity. This involves both prediction of de novo allergenicity as well as prediction of cross-reactivity. The latter, prediction of allergen cross-reactivity, is the topic of this communication, with emphasis on quantitative and methodological aspects.

Clinically, allergic cross-reactivity is often encountered as symptoms without prior exposure. Another common clinical situation is the occurrence of symptoms upon exposure to allergenic sources that are unlikely to sensitise, such as apples. In Northern Europe it is rare to find apple allergy in the absence of birch allergy. The major birch pollen allergen acts as the sensitizer or primary allergen, which by definition is able to trigger the immune system to produce IgE antibodies. The homologous protein in apple Mal d 1 is an incomplete allergen, because it is unable (or: extremely inefficient) to induce IgE antibodies, but is able to elicit symptoms due to its ability to trigger mast cells loaded with IgE anti-Bet v 1.

Cross-reactivity is sometimes seen as a property of a subgroup of antibodies: antibodies to some epitopes (recurring epitopes such as cross-reactive carbohydrate determinants (CCDs [2]) are more likely to be cross-reactive than antibodies to other epitopes. However, it is often more appropriate to use cross-reactivity to describe a relation between two allergens (which I will refer to as Ag1 and Ag2; alternatively, I will use the birch allergen Bet v 1 and the cross-reactive apple allergen Mal d 1 as examples): the closer the similarity between two allergens, the more likely it is to find a cross-reactive antibody. In either case, the concept of cross-reactivity concerns (at least) three rather than two reagents: two allergens and an antibody. Since it is impossible to test all antibodies, we have to live with the frustrating thought that it is impossible to prove that two allergens completely lack cross-reactivity. Conversely, it is also impossible to prove that they are fully cross-reactive. It is all a matter of probability, which in many cases is either very close to 0 or very close to 1. In many other cases it is, however, easy to demonstrate some cross-reactivity.

## Prediction of cross-reactivity from the amino acid sequence

Antibodies largely bind to surface patches of folded proteins (epitopes), so knowledge of the full 3D structures of the target protein and the related allergens would clearly be an advantage for such a prediction and now more commonly available (or the 3D structures can be reliably predicted). Undoubtedly, cross-reactivity prediction algorithms will be developed in which such information is incorporated. However, sufficiently reliable information on the relevant 3D structures is often not available and the prediction has to be based on the linear amino acid sequence. One of the points of debate is whether short stretches of 6–8 fully identical amino acids are reliable predictors and should be used in combination with partial amino acid identity of longer stretches (typically more than 35% identity between stretches of 80 amino acids [3]).

Other issues related to prediction of cross-reactivity, such as the repertoire-modifying effect of a human homologue, the contribution of post-translational modification (particularly non-mammalian glycosylation patterns), the possibilities and limitations of peptides as epitope mimics and the intriguing question whether IgE antibodies tend to be more cross-reactive than IgG antibodies, with its possible link with positive and negative regulation of B cells by IgE versus IgG antibodies, have been discussed elsewhere [4-6].

## Symmetric versus asymmetric cross-reactivity

**Figure 1:**
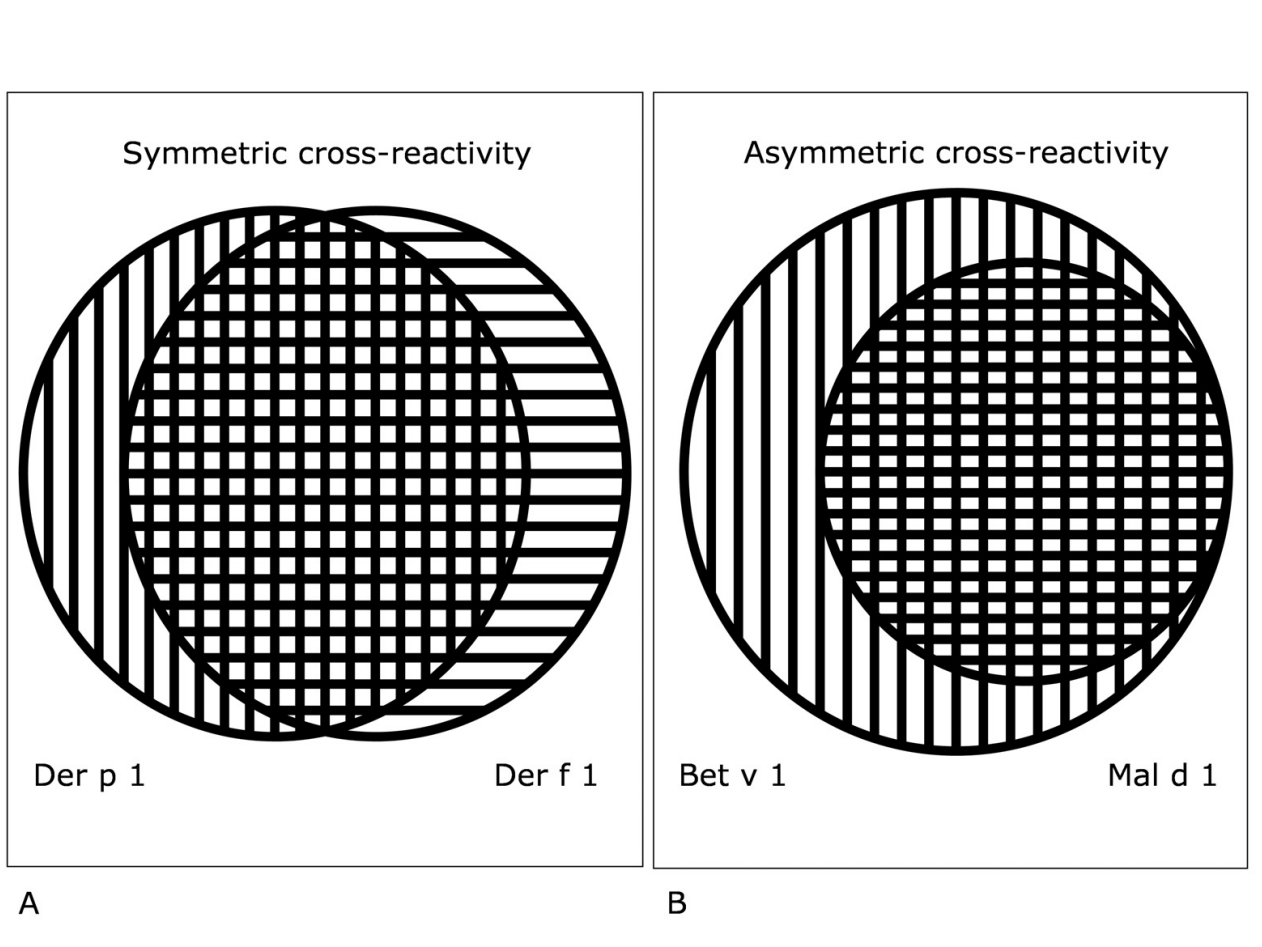
Symmetric (figure 1A) versus asymmetric (figure 1B) cross-reactivity. Symmetric cross-reactivity (figure 1A) occurs between major allergens from related grasses or dust mites. Asymmetric cross-reactivity (figure 1B) is usually found for example between the birch allergen Bet v 1 (outer circle) and the related protein from apple, Mal d 1 (inner circle). Bet v 1 completely inhibits IgE binding to Mal d 1, but Mal d 1 only partially inhibits IgE binding to Bet v 1 (see also figures 2 and 3).

Some situations of allergen cross-reactivity are almost trivial, such as the cross-reactivity between major allergens of botanically-related grasses [7] and between major dust mite allergens. Without information on allergen exposure it is then virtually impossible to decide which allergen is the sensitizer. Symmetric cross-reactivity a likely possibility: both allergens in the couple can sensitize and both can largely (but not completely) inhibit the binding of IgE to the other allergen (figure 1A). In the birch/apple situation the situation is different (at least in Northern Europe) [8]. Cross-reactivity is asymmetric (figure 1B), as can be demonstrated in vitro by reciprocal IgE antibody neutralization. The usual finding is that birch allergen inhibits IgE binding to the apple allergen similar to or even better than the inhibition found by using equimolar amounts of the apple allergen as inhibitor, whereas the apple allergen only partially inhibits IgE binding to the birch allergen.

## Issues on the quantification of the degree of cross-reactivity of two allergens

First, a semantic issue: polyclonal versus monoclonal cross-reactivity. The word "cross-reactivity" is used differently in the polyclonal situation and the monoclonal situation. Traditionally, it is used to describe the *polyclonal* situation as encountered in the body. Many different antibodies recognize Ag1 (for example: Bet v 1). Some of these antibodies react with Ag2 (Mal d 1). The degree of cross-reactivity can be expressed as the fraction of anti-Bet v 1 antibodies that react with Mal d 1. This fraction will vary between individuals and usually varies within one individual in time. A cross-reactive polyclonal antiserum can often be made mono-specific by absorption with either Ag1 or Ag2. When this type of cross-reactivity assessment is applied to a single *monoclonal* antibody, the unavoidable outcome would seem to be either: "fully cross-reactive" or: "not cross-reactive". This is intuitively unsatisfactory, since "fully cross-reactive" suggests equal reactivity of the antibody with Ag1 and Ag2, whereas in most cases the antibody will have different affinities for, and thus preferentially react with, either Ag1 or Ag2. An important qualitative modifier (which is mostly not specified in papers on cross-reactivity) is the affinity threshold, which depends on the read-out system and the concentrations used for testing. The effects of affinity are most visible in the monoclonal situation, but also play a role in the polyclonal situation. In general, a cross-reactive antigen will have a lower affinity than the antigen that induced the antibody response. It is not clear below which affinity the cross-reactivity becomes irrelevant, but it is important to appreciate that there is a grey area. In clinical terms, a low affinity may translate into a high threshold for the cross-reactive allergen and/or milder symptoms.

**Figure 2:**
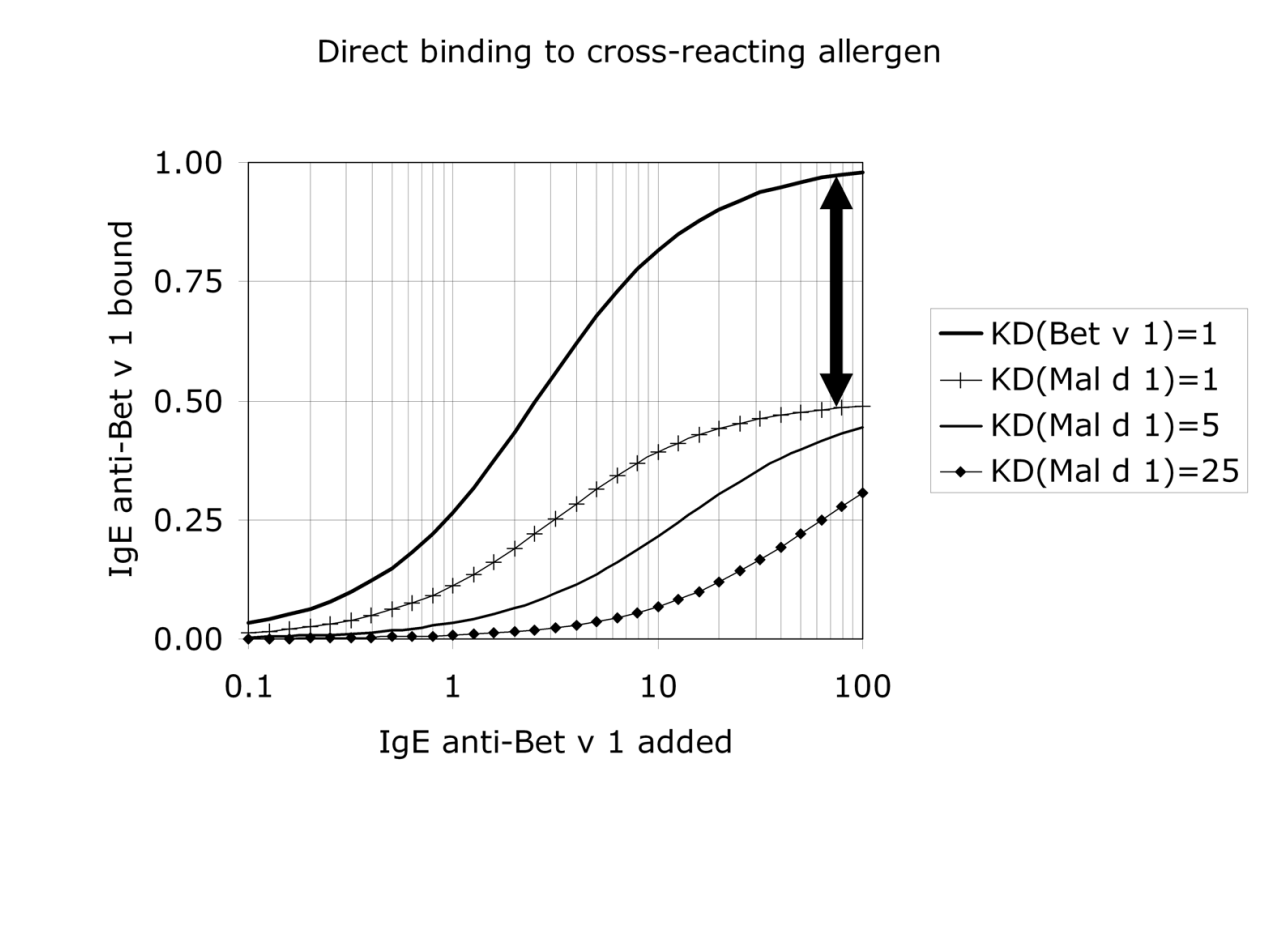
If a serum with cross-reactive antibody to the birch allergen Bet v 1 is incubated with Bet v 1 on the solid-phase, the binding curve is different in two ways: 1) it rises to a higher level and 2) it is shifted to the left compared to the binding observed with the cross-reactive apple allergen Mal d 1 on the solid phase. The first observation reflects that only a fraction of the epitopes is cross-reactive (in this example: 50%, as indicated by the vertical arrow). The second observation reflects that only a fraction of the antibodies is cross-reactive, but also that the affinity is usually lower for the cross-reactive allergen (Mal d 1) compared to the sensitising allergen (Bet v 1). In these model calculations, the concentration of Bet v 1 epitopes is set at 1; 50% of these epitopes are assumed have a cross-reactive homologue in Mal d 1; 40% of the IgE antibodies are assumed to be cross-reactive. The upper curve represents binding to Bet v 1, assuming a dissociation constant K_D _equal to 1. The next 3 curves represent binding to Mal d 1 at decreasing affinities (K_D _equal to 1, 5 and 25, respectively. Note that this type of experiment does not prove cross-reactivity. The observed binding could in theory also be due to co-sensitization. This can be investigated by inhibition assays (see figure 3).

**Figure 3:**
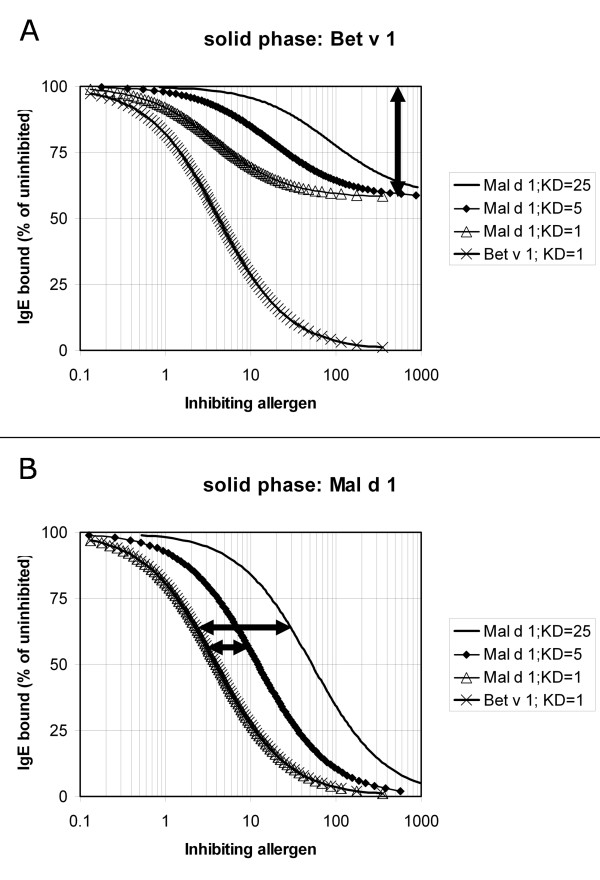
Model calculations illustrating the use of cross-inhibition to demonstrate cross-reactivity and to distinguish the primary sensitising birch allergen Bet v 1 from the cross-reactive (incomplete, non-sensitizing) apple allergen Mal d 1. The type of experiment shown in figure 3A indicates what percentage of the IgE antibodies is cross-reactive (in this example: 40%, as indicated by the vertical arrow). The type of experiment shown in figure 3B shows that Bet v 1 inhibits all antibodies to Mal d 1 and provides a crude estimate of the relative affinity of the interaction of the IgE antibodies with the two allergens (if the concentration of the antibody as well as the solid-phase allergen is equal to or below the K_D _of the IgE-allergen interaction), as indicated by the two horizontal arrows. The parameter setting correspond to those used in figure 2, using an IgE concentration of 1, which corresponds to values for uninhibited binding of IgE of 0.2660 for IgE binding to Bet v 1 in figure 3A (K_D _= 1), and for IgE binding to Mal d 1 of 0.0077, 0.0341 and 0.1118 (K_D _= 25, 5 or 1, respectively) in figure 3B.

In biological systems (e.g. skin test, cellular in vitro assays), assessment of cross-reactivity largely depends on direct testing, and thus on statistical associations. This is particularly unconvincing if not only the antibodies are polyclonal, but also the allergens are tested as allergen extracts (i.e. allergen mixtures). More reliable analysis is possible in vitro. In the case of monoclonal antibodies, direct binding assays can be used (figure 2). Also with polyclonal antibodies (typically from human serum) direct binding assays can be used and could, theoretically, provide information on one of the parameters in cross-reactivity assessment: the fraction of the epitopes that is cross-reactive. This is reflected in the relative IgE binding obtained at saturating IgE antibody doses (which corresponds to the vertical distance between the high-dose segments of two dose-response curves as indicated in figure 2). In practice, this is not a trivial analysis. Moreover, such a direct-binding test does not discriminate between cross-reacting and non-crossreacting IgE. Cross-reactivity can be proven (and to a certain extent quantified) by reciprocal inhibition systems, preferably with at least one purified single allergen (figure 3). The two other parameters in cross-reactivity assessment can be derived from such measurements: 1) the fraction of IgE that is cross-reactive (the vertical distance between the homologous and heterologous dose response curves when testing the complete allergen on the solid phase at saturating inhibitor levels as indicated in figure 3A) and 2) the relative affinity of the interaction between IgE and the two allergens (the horizontal distance between the homologous and heterologous dose response curves when testing the incomplete allergen on the solid phase, as indicated in figure 3B).

## A caveat on the use of serum pools

As discussed above, cross-reactivity is largely a probability feature. For this reason, it is rarely meaningful to test only one single serum. It is preferable to test a large number of individual sera and pool the results by conventional statistical procedures. However, the use of a serum pool rather than a large number of individual sera is, obviously, economical: fewer tests need to be performed and smaller volumes of (often precious) allergic serum samples are needed. From the statistical point of view it is important to inversely adjust the volumes of serum to be pooled according to their antibody titre: the higher the titre, the smaller to contributing volume should be. Optimally, the amount of antibody contributed by each serum donor should be similar (typically within a factor 2). Without such a precaution, it is likely that the pool will behave like a dilution of the strongest serum.
